# Biomarkers in COVID-19: An Up-To-Date Review

**DOI:** 10.3389/fped.2020.607647

**Published:** 2021-03-30

**Authors:** Madhusudan Samprathi, Muralidharan Jayashree

**Affiliations:** ^1^Rainbow Children's Hospital, Bannerghatta Road, Bangalore, India; ^2^Post Graduate Institute of Medical Education and Research, Chandigarh, India

**Keywords:** coronavirus disease 2019, severe acute respiratory syndrome by coronavirus 2, biomarkers, cytokine storm, laboratory investigations

## Abstract

The ongoing pandemic of coronavirus disease 2019 (COVID-19) poses several challenges to clinicians. Timely diagnosis and hospitalization, risk stratification, effective utilization of intensive care services, selection of appropriate therapies, monitoring and timely discharge are essential to save the maximum number of lives. Clinical assessment is indispensable, but laboratory markers, or biomarkers, can provide additional, objective information which can significantly impact these components of patient care. COVID-19 is not a localized respiratory infection but a multisystem disease caused by a diffuse systemic process involving a complex interplay of the immunological, inflammatory and coagulative cascades. The understanding of what the virus does to the body and how the body reacts to it has uncovered a gamut of potential biomarkers. This review discusses the different classes of biomarkers – immunological, inflammatory, coagulation, hematological, cardiac, biochemical and miscellaneous – in terms of their pathophysiological basis followed by the current evidence. Differences between children and adults are highlighted. The role of biomarkers in the diagnosis and management of Multisystem Inflammatory Syndrome in Children (MIS-C) is reviewed. The correlation of biomarkers with clinical and radiological features and the viral load, temporal evolution and the effect of treatment remain to be studied in detail. Which biomarker needs to be evaluated when and in whom, and how best this information can contribute to patient care are questions which currently lack convincing answers. With the evidence currently available broad guidelines on the rational use of available biomarkers are presented. Integrating clinical and laboratory data, monitoring trends rather than a single value, correlating with the natural course of the disease and tailoring guidelines to the individual patient and healthcare setting are essential.

## Introduction

The ongoing pandemic of severe acute respiratory syndrome by coronavirus 2 (SARS-CoV-2) continues to pose several diagnostic and therapeutic challenges. First reported from Wuhan in China in December 2019, the World Health Organization on February 11, 2020 officially named this infection, coronavirus disease 2019 (COVID-19) and the virus as SARS-CoV-2 (1). It was declared as a pandemic on March 11, 2020 (1). As on December 9, 2020, there are more than 67 million cases worldwide with more than 1.5 million deaths (2).

In adults, though SARS-CoV-2 typically causes pneumonia and acute respiratory distress syndrome (ARDS), it is now being recognized as a multisystem disease. In contrast, most children are asymptomatic or have mild to moderate illness. Severe or critical illness is rare (3, 4). A novel illness, termed multisystem inflammatory syndrome in children (MIS-C) is being increasingly reported in children. Children with MIS-C are sicker, may have multiorgan dysfunction and often require intensive care (5–8).

Diagnosis of COVID-19 is confirmed by direct detection of SARS-CoV-2 nucleic acids in respiratory tract specimens with a polymerase chain reaction (PCR) (9). A rapid and accurate diagnosis has wide implications for the patient, healthcare institution, and the public health and administrative personnel. In the current pandemic, healthcare systems are struggling to meet the increasing demands of the rapidly rising infected population. Effective utilization of available resources is paramount to saving the maximum number of lives. Clinical assessment is indispensable, but laboratory markers, or biomarkers, can provide additional, objective information which can significantly impact many components of patient care.

Despite the burgeoning COVID-19 literature database, the treating clinician needs to be effectively updated to offer the best care at the bedside. This review attempts to provide updated and practical information to clinicians on the role of biomarkers in COVID-19.

## Potential Role of Biomarkers in COVID-19

A biomarker is defined as a “characteristic that can be objectively measured and evaluated as an indicator of normal biological and pathological processes, or pharmacological responses to a therapeutic intervention” (10). Biomarkers in COVID 19 can be useful in the following areas:

- Early suspicion of disease
- Confirmation and classification of disease severity
- Framing hospital admission criteria
- Identification of high risk cohort
- Framing ICU admission criteria
- Rationalizing therapies
- Assessing response to therapies
- Predicting outcome
- Framing criteria for discharge from the ICU and/or the hospital

A strong working knowledge of the pathophysiology is essential for the initial identification of candidate biomarkers, which is, an understanding of what the virus does to the body and how the body reacts to it.

## Pathogenesis of COVID-19

### Overview

It is amply evident that COVID-19 is not a localized “*respiratory infection*” but a “*multisystem disease*” caused by a diffuse systemic process involving a complex interplay of the immunological, inflammatory and coagulative cascades. Genetic and acquired differences in the host immune system further complicate the host repertoire leading to wide heterogeneity in the clinical picture, course and outcome.

### Viral Entry and Replication

Coronaviruses are spheroidal, single-stranded RNA viruses with a diameter of 80–220 nm. Transmission of SARS-CoV-2 occurs either through exposure to micro-droplets from infected individuals or by contact transmission through contaminated fomites. The virus reaches the smaller airways and alveoli, and targets the bronchial and alveolar epithelial cells.

The spike surface glycoprotein S on the virus binds to angiotensin-converting enzyme 2 (ACE-2), a membrane carboxypeptidase present in distal airways and alveoli, especially type 2 pneumocytes which have the highest expression of ACE-2, along with alveolar macrophages and dendritic cells. ACE-2 is also expressed on the vascular endothelium, nasal, oral, nasopharyngeal, and oropharyngeal epithelia, gut epithelia, cardiac pericytes, renal proximal tubular cells and in the skin, reticuloendothelial and the central nervous system (11). ACE-2 expression depends on age, gender, genetic factors, and presence of comorbid conditions such as obesity, chronic cardiopulmonary disease, cancer, and use of immunosuppressive drugs.

Renin cleaves angiotensinogen to produce angiotensin I which is further cleaved by ACE to produce angiotensin II having a dual role. Action through AT1R (angiotensin II type 1 receptor), facilitates vasoconstriction, fibrotic remodeling, and inflammation, while that through AT2R (angiotensin II type 2 receptor) leads to vasodilation and growth inhibition. Angiotensin II is cleaved by ACE2 to Ang 1–7 which counteracts the harmful effects of the ACE/Ang II/AT1 axis. Thus ACE2 primarily plays a key role to physiologically counterbalance ACE and regulate angiotensin II. Internalization of the ACE-2 after viral interaction leads to its downregulation, and consequent upregulation of angiotensin II. The latter acting through AT1R, activates the downstream inflammatory pathways, leading to the “cytokine storm” that adversely affects multiple organs (12).

The alveolar epithelial cells, lymphocytes, and vascular endothelial cells are the primary targets of the virions. The virus inhibits the production of interferons which are part of cellular defense mechanisms. Viral replication releases a large number of virions leading to infection of neighboring target cells and viremia, which then cause an exaggerated pulmonary and systemic inflammatory response respectively. This explains the clinical presentation of severe COVID-19 which is predominated by ARDS, shock, and coagulopathy.

The immunological, inflammatory, and coagulative cascades are closely interlinked. Table 1 lists the various biomarkers according to the organ or system of origin, portraying the multisystem nature of COVID-19.

**Table 1:** List of biomarkers in COVID-19 classified according to organ/system involved.

Hematological	Leucocytosis/leucopenia Lymphopenia Neutrophilia Depletion of CD4+ and CD8+ cells Elevated neutrophil-to-lymphocyte ratio (NLR) Thrombocytopenia/thrombocytosis
Inflammation	Cytokines Chemokines Growth factors C reactive protein Procalcitonin Lactate dehydrogenase
Coagulation	D-dimer levels Fibrinogen Fibrin degradation products (FDP) Prothrombin time (PT) Activated partial thromboplastin time (aPTT)
Cardiac	Cardiac troponin (ctn) Brain natriuretic peptide (BNP)/NT-proBNP
Hepatic	Aspartate aminotransferase (AST) Alanine aminotransferase (ALT) Bilirubin Albumin
Muscle	Creatine-kinase (CK) Myoglobin
Renal	Serum creatinine
Electrolytes	Hyponatremia Hypokalemia Hypocalcemia

### Subsequent Events and Organ Damage

#### Immune Response and Inflammation

SARS-CoV-2 infection triggers both innate and adaptive immune response (13). Excessive pro-inflammatory response of the former and dysregulated host response of the latter leads to tissue damage. The ensuing widespread uncontrolled immune dysregulation releases massive amounts of cytokines and chemokines what is typically called the “cytokine storm.”

Both CD4+ and CD8+ T cells are antiviral. Of these, the latter account for about 80% of the total infiltrative inflammatory cells and can kill virus infected cells. The former on the other hand, activate the T-dependent B cells which produce virus specific antibodies. The balance between naïve and memory T cells is crucial for an efficient host defensive response (13). While naïve T cells are defense against new and previously unrecognized infection, memory T cells mediate antigen-specific immune response. An imbalance favoring naïve T cell activity as against regulatory T cells, contributes to hyperinflammation through a massive, coordinated release of cytokines. Significant changes are also seen in B cells.

The complement pathway also plays an important role in the hyperinflammation. C3a and C5a with potent pro-inflammatory properties, trigger inflammatory cell recruitment and neutrophil activation. Cytokine storm, the hallmark of SARS CoV2 infection, evolves through several pathways, like the NF-κB, JAK/STAT and the macrophage activation pathway, leading to the release of interleukin-6 (IL-6) and TNF-alpha (13). IL-6 is a key player in the cytokine storm, activating several cell types and forming a positive feedback loop. The large-scale unregulated production of interleukins, particularly IL-6, further stimulates several downstream pathways, increasing the production of acute-phase reactants like C-reactive protein (CRP).

#### The Coagulative Cascade and Escalating Inflammation

The coagulative cascade involves endothelial cells, platelets, neutrophils, monocytes, and macrophages. A healthy vascular endothelium is both anti-thrombotic and anti-inflammatory. This protective barrier is disrupted in COVID-19 leading to thrombosis and inflammation (14) primarily driven by thrombin (14).

Primary hemostasis begins with platelet activation. Platelets once activated recruit more platelets and crosslink with them via fibrinogen. Platelets secrete proinflammatory cytokines and proangiogenic factors like vascular endothelial growth factor (VEGF) and promote leukocyte activation and extravasation. The enhanced inflammatory state, thrombi formation and platelet consumption can lead to thrombocytopenia whilst the cytokine storm causes thrombocytosis.

Neutrophils recruited to growing thrombi form neutrophil extracellular traps (NET), the organized extrusion of the chromatin of mature neutrophils. NETs are anti-bacterial and prothrombotic.

Macrophages, recruited to fibrin thrombi, generate plasmin, through which fibrin is degraded to D-dimers. Thus macrophages possibly contribute to the unusually extreme elevation of D-dimers seen in COVID-19.

Activated monocytes and damage-associated molecular patterns from injured tissues produce inflammatory cytokines and chemokines which stimulate neutrophils, lymphocytes, platelets, vascular endothelial cells and monocytes to express tissue factor and phosphatidylserine and trigger coagulation.

Autopsy analyses have revealed fibrin-rich thrombi containing neutrophils in the alveolar capillaries and increased lung megakaryocytes producing young platelets which are more thrombogenic (14). Owing to the high fibrinolytic capacity of lungs, there is vigorous fibrinolysis leading to the production of D-dimers which spill into the blood.

#### Lymphocytopenia

Lymphocytopenia, a hallmark of COVID-19, is attributed to multiple mechanisms including (i) direct viral invasion and lysis as lymphocytes express the ACE2 receptor on their surface (ii) lymphocyte apoptosis induced by interleukins (iii) reduced lymphocyte turnover due to the “cytokine storm” induced atrophy of lymphoid organs, and (iv) reduced lymphocyte proliferation due to lactic acidosis (15).

#### Cardiac Injury

Cardiac injury plays an important role in disease progression and outcome. Direct viral infection and damage and immune mediated damage are the two mechanisms proposed for cardiac injury (16). Viral infection of cardiomyocytes and intracellular replication leads to cardiomyocyte degeneration and necrosis, causing cardiac dysfunction and arrhythmia. The immune-mediated mechanism involves the cytokine storm leading to microcirculation defects, tissue ischemia, and hypoxia. The pro-inflammatory state is also said to aggravate atherosclerosis and immune complex precipitation which can increase the possibility of acute myocardial infarction.

#### Pathogenesis of MIS-C

The pathogenesis of MIS-C is not completely understood. It is suspected to coincide with the development of acquired immune response to the virus, rather than direct viral invasion. Antibody responses in children and adults have been found to be markedly different. Some of the postulated mechanisms include antibody-dependent enhancement of viral entry and replication, immune complex mediated triggering of the host inflammatory response or direct anti-tissue antibody activation or cellular activation, or both (17). Multiple autoantibodies are suspected to be involved. The inflammatory response in MIS-C is different from the hypercytokinemia seen in severe acute COVID-19. Although its clinical features overlap with that of Kawasaki disease, it differs from this condition with respect to T cell subsets, interleukin (IL)-17A, and biomarkers associated with arterial damage (18).

## Biomarkers Studied in COVID-19

In the following section, we discuss how the above knowledge on pathophysiology can translate into practice with the laboratory tests available to the clinician.

### Hematological Parameters

#### Hemoglobin

In a retrospective study, anemia and altered iron homeostasis were common in hospitalized COVID-19 patients. Initial anemia was associated with increased mortality, and a higher ferritin/transferrin ratio predicted need for ICU admission and mechanical ventilation (19).

#### Lymphocytes

Peripheral blood leukocyte and lymphocyte counts are normal or slightly reduced in early disease, when symptoms tend to be non-specific (15). Approximately 7 to 14 days from the onset of symptoms, appearance of significant lymphopenia coincides with worsening clinical status, increase in inflammatory mediators and “cytokine storm.”

Lymphocytopenia is directly correlated with disease severity and death. Lymphopenia on admission (defined as lymphocyte count ≤ 1,100 cells/μl) is associated with three-fold risk of poor outcome, in younger as compared to older patients (15). Lymphocyte counts were lower in patients with ARDS, severe disease requiring ICU care, and in non-survivors (20). A temporal model based on lymphocyte counts at two time points showed that patients with <20% and <5% lymphocytes at days 10–12 and 17–19 from the onset of symptoms respectively had the worst prognosis (21).

Severe disease was also characterized by marked reduction in the absolute number of circulating CD4+ cells, CD8+ cells, B cells and natural killers (NK) cells.; plasma cells are remarkably increased (13, 22).

The highest values of inflammatory parameters firmly correlated with the decrease in CD8 T-cells, an effect that was not seen with CD4 cells (23).

#### Neutrophils

Patients requiring admission to the ICU had higher percentage and absolute number of neutrophils (23).

#### Eosinophils

A low percentage of eosinophils and airway and serum eosinophil-derived neurotoxin (EDN-1) can be a potential biomarker of COVID pneumonia (24, 25). However further studies are required to correlate EDN-1 with clinical, radiographic, and physiological parameters (25).

#### Monocytes and Basophils

Monocytes and basophils are also decreased akin to lymphocytes and eosinophils.

#### Platelets

Both thrombocytopenia and thrombocytosis have been observed. However severe thrombocytopenia and bleeding are uncommon (26). Thrombocytopenia was shown to correlate with other coagulation parameters and increased risk of mortality (27).

#### Composite Hematological Markers

Putting it all together it is clear that severe COVID 19 disease is associated with significantly increased leukocytes, neutrophils, infection biomarkers [such as CRP, PCT and ferritin] and cytokine levels [IL-2R, IL-6, IL-8, IL-10 and tumor necrosis factor (TNF)-α] and decreased lymphocyte counts (28).

IL-2R levels correlated positively with the other cytokines and negatively with lymphocyte number. An elevated IL-2R to lymphocytes ratio was discriminative of severe and critical illness. In fact this ratio was superior to other markers for differentiation of critical illness. The ratio was significantly decreased in recovered patients, but further increased in patients who deteriorated, thus correlating with the outcome (28).

Zheng et al. devised a score based on the neutrophil, lymphocyte and platelet counts, with an “NLP score” of >6, predicting progression to severe disease (29). A high neutrophil-to-lymphocyte ratio (NLR) at admission can be a good surrogate marker for diagnosis of COVID-19. A rising NLR can also be used as a prognostic marker for predicting poor outcomes. (30, 31). Another prognostic marker the lymphocyte-to-CRP ratio (LCR), used in several types of cancers, may also be helpful. A meta-analysis on six studies concluded that a rise in the NLR and decline in LCR correlates with the severity of COVID-19 (32). Specifically, a low LCR at presentation was seen to predict ICU admission and need for invasive ventilation.

### Inflammatory Parameters

#### CRP and Procalcitonin

In a study, CRP was elevated in 60.7% of patients, procalcitonin (PCT) in 5.5%, and lactate dehydrogenase (LDH) in 41% of patients (33). A cut off of >10 mg/L and >0.5 ng/ml for CRP and PCT respectively have been shown to be predictors of poor outcome (34). A retrospective study showed that a CRP level of 26 mg/L could serve as a cut-off to predict progression to severe disease (35). A meta-analysis showed that elevated PCT values were associated with a nearly 5-fold higher risk of severe infection (36).

#### Cytokines

IL-6 is dramatically increased in COVID-19 patients. More than half of admitted patients were found to have elevated IL-6 levels (37). Higher baseline IL-6 correlated with severity, bilateral interstitial lung involvement and other acute inflammatory markers (38). Several meta-analyses and systematic reviews have identified IL-6 as an important marker of disease severity and predictor of mortality (Table 2). Furthermore, IL 6 is good to monitor therapeutic response. Other pro-inflammatory cytokines (IL-1β, IL-2, IL-8, IL-17, G-CSF, GMCSF, IP-10, MCP-1, CCL3, and TNFα) are significantly increased in patients with severe disease (12, 13).

**Table 2:** Summary of the meta-analyses and systematic reviews investigating the role of biomarkers in COVID-19.

**Authors**	**Objective**	**Studies included**	**Findings**
Zeng et al. (39)	Associations of inflammatory markers with the severity of COVID-19	16 studies 3,962 patients (adults)	Patients in the non-severe group had lower levels of CRP, PCT, IL-6, ESR, SAA and serum ferritin compared with those in the severe group. Survivors had a lower level of IL-6 than non-survivors.
Wu et al. (40)	To identify variables in detecting severe COVID-19	41 studies 5,064 patients	There was significant increase in WBC, CRP, D-dimer, AST and LDH in the severe cases.
Zhu et al. (41)	To analyse coagulation parameters of COVID-19 patients in order to provide a reference for clinical practice.	34 studies 6,492 patients	Patients with severe disease showed significantly lower platelet count and shorter activated partial thromboplastin time but higher D-dimer levels, higher fibrinogen levels and longer prothrombin time. Patients who died showed significantly higher D-dimer levels, longer prothrombin time and lower platelet count than patients who survived.
Lin et al. (42)	Meta-analysis and systematic review of blood coagulation indicators in patients with severe COVID-19	13 studies 1,341 adult patients	Platelet, d-dimer and fibrinogen were significantly associated with severity. No correlation was evident between an increased severity risk and activated partial thromboplastin time or prothrombin time. Coagulation dysfunction is closely related to the severity of patients with COVID-19, in which low platelet, high d-dimer, and fibrinogen upon admission may serve as risk indicators for increased aggression of the disease.
Tian et al. (43)	To evaluate the risk factors associated with mortality	14 studies 4,659 patients	Non-survivors, compared to survivors, had elevated levels of cardiac troponin, CRP, IL-6, D-dimer, creatinine and ALT as well as decreased levels of albumin.
Figliozzi et al. (44)	Identification of reliable outcome predictors	49 studies were selected for a pooled assessment	Advanced age, male sex, cardiovascular comorbidities, acute cardiac or kidney injury, lymphocytopenia and D-dimer conferred an increased risk of in-hospital death.
Alnor et al. (45)	To examine the laboratory tests are associated with severe COVID-19 disease.	45 studies, 21 publications used for meta-analysis	Severe disease was associated with higher WBC count, neutrophil count, CRP, LDH, D-dimer, AST, and lower platelet count and hemoglobin.
Ghahramani et al. (46)	To compare the laboratory test findings in severe vs. non-severe confirmed cases of COVID-19.	22 studies 3396 patients	A significant decrease in lymphocyte, monocyte, and eosinophil, hemoglobin, platelet, albumin, serum sodium, lymphocyte to C-reactive protein ratio (LCR), leukocyte to C-reactive protein ratio (LeCR), leukocyte to IL-6 ratio (LeIR), and an increase in the neutrophil, ALT, AST, total bilirubin, blood urea nitrogen, creatinine, ESR, CRP, PCT, LDH, fibrinogen, prothrombin time, D-dimer, glucose level, and neutrophil to lymphocyte ratio (NLR) were seen in the severe group compared with the non-severe group.
Di Minno et al. (47)	Association of COVID-19 severity with changes in hemostatic parameters.	60 studies 5,487 subjects with severe and 9670 subjects with mild COVID-19	Documented higher PT, D-Dimer, and fibrinogen values, with lower platelet count among severe patients. Twenty-five studies on 1511 COVID-19 non-survivors and 6287 survivors showed higher prothrombin time and D-Dimer values, with lower platelet count among non-survivors. Regression models showed that CRP values were directly correlated with the difference in prothrombin time and fibrinogen.
Henry BM et al. (48)	To evaluate the discriminative ability of hematologic, biochemical and immunologic biomarkers in patients with and without the severe or fatal forms of COVID-19.	21 studies 3,377 patients 33 laboratory parameters	Patients with severe and fatal disease had significantly increased WBC count, and decreased lymphocyte and platelet counts compared to non-severe disease and survivors. Biomarkers of inflammation, cardiac and muscle injury, liver and kidney function and coagulation measures were also significantly elevated in patients with both severe and fatal COVID-19. IL-6 and IL-10 and serum ferritin were strong discriminators for severe disease.
Pranata et al. (49)	To assess the association between N-terminal pro-brain natriuretic peptide (NT-proBNP) and mortality in patients with COVID-19	6 studies 967 patients	Elevated NT-proBNP level was associated with increased mortality in COVID-19 pneumonia.
Soraya et al. (50)	To determine the differences between laboratory parameters in (1) COVID-19 versus non-COVID-19 pneumonia, and (2) severe versus non-severe COVID-19 cases.	1^st^ analysis: 7 studies2^nd^ analysis: 26 studies	Seven studies in the first analysis showed significantly lower leukocyte, neutrophil and platelet counts in COVID-19 pneumonia compared to non-COVID-19 pneumonia. Twenty-six studies in the second analysis showed significantly lower lymphocyte and thrombocyte counts and significantly higher leukocyte, neutrophil, D-dimer, and CRP in severe COVID-19 compared to non-severe COVID-19.
Huang et al. (34)	To investigate the association between several biomarkers, including serum CRP, PCT, D-dimer, and serum ferritin, and COVID-19 severity	25 studies 5,350 patients	Elevated CRP was associated with poor outcome. A CRP ≥10 mg/L had 51% sensitivity, 88% specificity, likelihood ratio (LR) + of 4.1, LR- of 0.5, and an area under curve (AUC) of 0.84. An elevated PCT was associated with poor outcome and mortality. A PCT ≥0.5 ng/ml had 88% sensitivity, 68% specificity, LR+ of 2.7, LR- of 0.2, and an AUC of 0.88. Elevated D-dimer was associated with mortality. A D-dimer >0.5 mg/L has 58% sensitivity, 69% specificity, LR+ of 1.8, LR- of 0.6, and an AUC of 0.69. Patients with a composite poor outcome had a higher serum ferritin.
Toriah et al. (51)	To systematically explore the association of COVID-19 severity and mortality rate with the history of cardiovascular diseases and/or other comorbidities and cardiac injury laboratory markers	A meta-analysis of 17,794 patients	Patients with high cardiac troponin I and AST levels were more likely to develop adverse outcomes. High troponin I >13.75 ng/L combined with either advanced age >60 years or elevated AST level >27.72 U/L was the best model to predict poor outcomes.

*ALT, alanine transaminase; AST, aspartate transaminase; CRP, C-reactive protein; ESR, erythrocyte sedimentation rate; IL-6, interleukin-6; LDH, lactate dehydrogenase; NT-proBNP, N terminal pro brain natriuretic peptide; PCT, procalcitonin; SAA, serum amyloid A; WBC, white blood cell*.

A small study showed that cytokine levels in COVID-19 patients with ARDS were lower than in those with septic shock, trauma or out of hospital cardiac arrest, commensurate with lower leucocyte counts (52). These preliminary findings question the existence of cytokine storm and benefit of anticytokine therapies.

#### Ferritin

Studies on ferritin levels in COVID-19 patients have yielded equivocal results. It is not clear whether it is a bystander or a true characteristic of the disease (53). Two retrospective studies have reported minimal role of ferritin in predicting ICU admission and need for ventilation and failure in predicting mortality (19, 54). But another study and a meta-analysis showed findings to the contrary; ferritin levels could predict severe disease and mortality (34, 54).

### Coagulative Parameters

Coagulopathy in COVID-19 differs from the usual disseminated intravascular coagulation, in having a high fibrinogen, normal or mildly prolonged prothrombin time and activated partial thromboplastin time, platelet count >100 × 10^3^/ml, but no significant bleeding (14).

Elevated D-dimer levels are very frequently seen in patients with COVID-19. Several meta-analyses have shown that D-dimer levels have prognostic value and correlate with disease severity and in-hospital mortality (Table 2). A level of >2.0μg/ml on admission could predict mortality (55, 56). D-dimer can be an early marker to guide management of Covid-19 patients.

### Cardiac Biomarkers

Cardiac biomarkers have been studied in diagnosis, triaging, treatment, and prognosis. Raised cardiac biomarkers including LDH, creatine kinase (CK), creatinine kinase-muscle/-brain activity (CK-MB), myoglobin (Mb), cardiac troponin I (cTnI), alpha-hydroxybutyrate dehydrogenase (α-HBDH), aspartate aminotransferase (AST), and N-terminal of the prohormone brain natriuretic peptide (NT-proBNP) have been seen in patients with COVID-19. Among these LDH, CK, α-HBDH, and AST are not myocardial specific and may be elevated in injury to lungs, liver and kidneys.

On the other hand, CK-MB, cTnI, Mb, and NT-proBNP are more myocardial injury specific and increased to varying degrees, especially in severe and critical illness. Furthermore, higher levels were associated with higher mortality (57, 58). Cut-offs of these biomarkers to predict mortality have been found to be much lower than for regular heart disease (59). Troponin and natriuretic peptides have been studied for risk stratification, to aid decision making with regard to rational use of ECG/echocardiography and aggressive therapies, and prognostication (60).

Cardiac biomarkers have been seen to be in tandem with other biomarkers; patients with myocardial injury had higher leukocyte, lower lymphocyte and lower platelet counts (48).

However, cardiac biomarkers need to be used judiciously as routine tests in all patients may be misleading.

### Biochemical Parameters

#### Serum Albumin

Hypoalbuminemia in critically ill patients is multifactorial and is attributed to increased capillary permeability, decreased protein synthesis, increased turnover, decreased serum albumin total mass, increased volume of distribution, and increased expression of vascular endothelial growth factor. Although common, the exact temporal association of hypoalbuminemia is yet to be studied (61).

In COVID 19 disease a similar trend was found; a meta-analysis of 11 studies showed that the mean serum albumin on admission was 3.50 g/dl and 4.05 g/dl in severe and non-severe COVID-19, respectively (61).

#### LDH

About 40% of patients presented with increased LDH levels. Elevated LDH has been associated with a higher risk of ARDS, need for intensive care and mortality (15).

#### Urine Biochemical Parameters

Urine biochemical parameters have been studied to predict the severity of disease. The positive rates of urine glucose and protein were higher in the severe and critical groups compared to those in the moderate group. Urine occult blood and specific gravity were not associated with the severity (62).

Table 2 summarizes the meta-analyses and systematic reviews investigating the role of various biomarkers in COVID-19.

## Comparison of Children and Adults

Children, fortunately are mostly asymptomatic or have a mild illness, compared to adults. In a meta-analysis on children, 20% were asymptomatic; 33% had a mild illness and 51%, a moderate illness (4). Critical cases were seen in 14% of infants. Another Chinese study reported severe and critical cases in 10.6, 7.3, 4.2, 4.1 and 3.0% for the age group of <1, 1–5, 6–10, 11–15, and >15 years, respectively (63).

Overall, laboratory abnormalities are uncommon in children and are predominantly confined to children with severe disease and MIS-C (64–67).

Similar to adults, abnormal laboratory markers reported include serum D-dimer, PCT, creatine kinase, and IL-6 (68). The most commonly reported abnormal laboratory parameters in children were leukopenia/lymphopenia (16–33%), leukocytosis (13%), increased creatine kinase (5–37%), elevated D-dimer (12–52%), CRP (17–40%), AST (19%) and alanine aminotransferase (15%) (4, 63, 68–70).

Children with severe respiratory disease and MIS-C have significantly higher CRP, procalcitonin, or troponin level, and a lower nadir ALC, platelet count, or serum sodium level compared to those with non-severe disease (66, 71). Children requiring intensive care were more likely to have lower platelet counts, higher neutrophil counts and higher CRP, but these differences were less marked when children with MIS-C were excluded (72).

Serum interleukin-17A and interferon-⋎ (IFN- ⋎), but not tumor necrosis factor– α (TNF-α) or IL-6, were inversely related to age. Neutralizing antibody titers correlated positively with age and negatively with IL-17A and IFN- ⋎ serum concentrations (71).

Laboratory biomarkers play a major role in the diagnosis, prognosis and management of children with MIS-C. Other than fever, which is universal, rest of the clinical manifestations are present in a variable percentage of patients. Hence, biomarkers are a valuable adjunct in timely diagnosis; appropriate therapy is lifesaving. Several diagnostic criteria have been proposed (73). Mandatory investigations for all children with suspected MIS-C, which are an essential part of the WHO criteria are the (i) inflammatory markers – ESR, C-reactive protein, or procalcitonin, and ferritin, (ii) cardiac biomarkers – troponin/NT-proBNP, and (iii) markers of coagulopathy – PT, PTT, and d-dimers. Elevated fibrinogen, LDH, or IL-6, elevated neutrophils, reduced lymphocytes and low albumin, proteinuria, high CK, high TG, and transaminitis are present in a variable number of children.

A systematic review on children with MIS-C reported neutrophilia in 83% of cases, raised CRP in 94%, lymphopenia in 50%, raised Troponin-T in 68% and raised proBNP in 77% of cases (74). With majority of patients having a high CRP, and values higher than 100 mg/L being common, CRP is a valuable, inexpensive initial investigation to screen patients for MIS-C as well as to monitor them after therapy (74, 75).

## Temporal Trends in Biomarkers

As detailed above, there are numerous individual studies and several meta-analysis on many potential biomarkers. Most of them mainly display the differential change in biomarkers between disease categories and relation to outcomes like mortality, need for ICU admission, mechanical ventilation and duration of hospital stay.

However, the temporal variation of biomarkers along the course of the illness is important to ascertain disease progression and therapeutic response.

A retrospective study from Wuhan, China during the early pandemic showed that WBC and neutrophil counts were normal in the first week and increased subsequently (76). Lymphopenia, more prominent in non-survivors, however persisted throughout in all patients. Thrombocytopenia noted in the first week improved subsequently in survivors and persisted in non-survivors. D-dimer level was elevated in non-survivors later in the illness. Non-survivors as compared to survivors had higher levels of CK, CK-MB, LDH, AST, and ALT in the early part of the illness, and progressively rising blood urea and creatinine levels.

Another retrospective study followed the trend of blood counts along the course of the disease (77). In severe/critical cases, WBC, neutrophil and platelet counts progressively fell to a nadir by day 8–9 of illness but gradually recovered in the subsequent days. The lymphocyte count decreased gradually, the proportion of reactive and antibody synthesizing lymphocytes progressively increased toward day 15–16.

Hematological and immunological parameters assessed over time showed that lymphocytes, T-cell subsets, eosinophils, and platelets were markedly low at admission, especially in severe/critical disease and non-survivors. Survivors and non-survivors could be discriminated by increasing trend of eosinophils, lymphocytes, and platelets in the former compared to a significant drop in the latter. Restored levels of lymphocytes, eosinophils, and platelets could serve as predictors for recovery, whereas progressive increases in neutrophils, basophils, and IL-6 were associated with fatal outcome (37).

A small retrospective study revealed that ferritin was the last parameter to return to normal while high-sensitivity CRP, normalized about 5 days before ferritin (78) thus suggesting that ferritin is more useful in assessment of the severity, rather than monitoring the course of the disease.

A prospective study analyzed key immune mediators temporally over 4 weeks (79). MCP-1 and the inhibitory cytokines, IL-1RA and IL-10, were higher in severe cases in the first 2 weeks as compared to mild cases but not in the subsequent 2 weeks of illness. IL-6, IL-17, IL-12, IL-1β, IFN-γ, and IL-27 were elevated in severe cases about 4 weeks after symptom onset. RANTES, also called CCL5, was elevated in mild but not severe cases throughout the first month of illness. Taken together, a combination of CCL5, IL-1RA, and IL-10 may be useful to predict patient outcomes in the first week of illness.

A study, classifying patients in 3 categories (mild, severe and fatal), estimated levels of 48 biomarkers (cytokines, chemokines and growth factors – CCGF) serially on days, 1, 5, 10, and 14 after diagnosis (80). Twelve of the CCGFs (including IFN-γ, IL-1Rα, IL-2, IL-2Rα, IL-6) were upregulated to similar levels on days 1 and 5 in all three categories, but were markedly further upregulated in fatal patients on day 14 after diagnosis, while remaining at steady levels in survivors.

Table 3 summarizes the temporal course of various biomarkers.

**Table 3:** Temporal course of biomarkers in COVID-19.

**Time period**	**Biomarkers**
<7 days	• Total leucocyte count & lymphocyte count normal or slightly low • ↑ LDH, ↑ AST, ↑ ALT, ↑ CK, ↑ CK-MB – may be early markers of severe disease and mortality
7–14 days	• Total leucocyte count & lymphocyte count progressively fall to reach nadir at 8–9 days • Thrombocytopenia may occur • ↑ IL-6, IL-10, IL-1RA, MCP-1
>14 days	• Increasing total leucocyte count, lymphocyte & platelet count predict recovery while reducing counts predict mortality

*ALT, alanine transaminase; AST, aspartate transaminase; CK, creatine kinase; IL-6, interleukin-6; LDH, lactate dehydrogenase; MCP, monocyte chemoattractant protein 1*.

## Association of Biomarkers With Clinical Phenotype and Therapeutic Response

COVID, like ARDS of any other etiology, is a spectrum of varying phenotypes where customized therapy may help more and harm less. Rello et al. have described 5 phenotypes ranging from the most benign (phenotype 1) to increasing respiratory distress and hypoxemia (phenotypes 2 and 3) and ARDS (phenotypes 4 and 5) (81). IL-6 has been suggested as a differentiating feature between phenotypes 2 and 3, and PCT as a characteristic feature of phenotype 5. Defining phenotypes based on underlying risk factors, clinical and radiological features and biomarkers may help in predicting need for ICU and optimizing therapy.

The correlation of biomarkers with clinical and radiological features and the viral load, and the effect of treatment remain to be studied in detail. None of the proposed anti-viral, anti-inflammatory, anticoagulant and anti-fibrotic therapeutic strategies have been proven to be conclusively beneficial.

One study assessed the changes in biomarkers with supportive therapy and a variable combination of abidol, lopinavir/ritonavir and methylprednisolone (82). After treatment, IL-2R, IL-6, TNF-α, and CRP levels decreased significantly, followed by IL-8, IL-10, and PCT. CD4+ and CD8+ T lymphocytes increased significantly but B lymphocytes and natural killer cells showed no changes. Serum ferritin also did not decrease significantly.

D-dimer levels have been recommended as a part of the risk stratification criteria to decide anticoagulation (83).Treatment with low molecular weight heparin (LMWH) is associated with reduction in levels of d-dimer and fibrin degradation products and also in IL-6 levels suggesting a potential anti-inflammatory effect (84).

Tocilizumab has been considered for patients with severe and extensive lung disease with elevated IL-6 levels but evidence is conflicting (85–87).

## Bench to Bedside

As the pandemic is evolving, strategies for testing, treatment and control are changing and will vary across countries and regions. Which biomarker needs to be evaluated when and in whom, and how best this information can contribute to patient care are questions which currently lack convincing answers. Table 4 summarizes the role of biomarkers in the diagnosis and management of patients with COVID-19.

**Table 4:** Role of biomarkers in various areas of patient management.

**Specific role**	**Biomarkers**
Diagnosis	Leukopenia Lymphopenia High NLR ratio ↑ LDH ↑ AST
Assessment of severity	Lymphopenia Lymphocyte subsets - ↓CD4+, CD8+, B, NK cells, ↑ plasma cells ↑ NLR & ↓ LCR ↑ IL-2R/Lymphocytes ratio ↑ IL-6 ↑ CRP, PCT ↑ Ferritin ↑ LDH ↑ D-dimers ↑ Specific cardiac biomarkers – CK-MB, CTnT, Mb, NT-proBNP
Response to therapy	↓ CRP ↓ IL-6, IL-10, TNF-alpha, IL-2R
Prognosis	IL-6 Ferritin LDH CRP, PCT Lymphocyte count NLR LCR Platelet count Specific cardiac biomarkers – CK-MB, CTnT, Mb, NT-proBNP
MIS-C	CBC - ↓ platelets ↑ CRP, ESR, PCT ↓ Albumin ↑ Specific cardiac biomarkers – CK-MB, CTnT, NT-proBNP ↑ IL-6, IL-10, TNF-alpha ↑ D-dimers

*ALT, alanine transaminase; AST, aspartate transaminase; CK, creatine kinase; CRP, C-reactive protein; CT, computed tomography; CTnT, cardiac troponin-T; ESR, erythrocyte sedimentation rate; IL-6, interleukin-6; LCR, lymphocyte to CRP ratio; LDH, lactate dehydrogenase; NLR, neutrophil to lymphocyte ratio; NT-proBNP, N terminal pro brain natriuretic peptide; PCT, procalcitonin; SAA, serum amyloid A; TNF-alpha, tumor necrosis factor-alpha; WBC, white blood cell*.

### General Principles

- Diagnosis, especially in the first week of illness, will be established by using the standard clinical case definitions and molecular testing with RT-PCR.
- COVID-19, especially MIS-C, can have a wide spectrum of presentation and needs to be considered in any acute febrile illness, especially but not necessarily with abdominal, and respiratory symptoms.
- Leukopenia, lymphopenia, a high NLR ratio, raised LDH and AST levels may help in differentiating COVID-19 from other similar illnesses.
- It is important to stratify patients based on severity determined by clinical and laboratory parameters. Multiple biomarkers have been seen to predict the severity of disease and have been listed in Table 3.
- Serial trends rather than single measurements combined with clinical assessment is the best method in deciding the COVID 19 specific treatment modalities.
- Biomarkers that predict a poor prognosis are listed in Tables 3 and 4. It is important to consider the timing of testing and the trends to obtain a meaningful picture.

### General Recommendations

Based on the currently available evidence, summarized in Tables 3 and 4, we would like to offer the following recommendations with respect to the use of biomarkers in adults and children with COVID-19, including children with MIS-C.

These are primarily based on the clinical categorization as per the WHO guidelines (88), but should be modified according to the clinical condition, presence of comorbidities, availability and cost.

- For patients who are asymptomatic or in the mild category (without underlying comorbidity), no investigations are needed.
- For all patients in the mild category with associated comorbidity or patients in the moderate category, a complete blood count (CBC), CRP, serum creatinine, and liver function tests are needed at admission. If any of these markers are abnormal, further investigations mentioned for patients in the severe category may be considered. If symptoms persist in the second week, CBC and CRP must be repeated to see the trends to decide monitoring and further investigations.
- For all patients in the severe category, in addition to the markers mentioned above, PT, APTT, INR, serum ferritin, d-dimer and cardiac biomarkers (NT-pro-BNP and troponin I) are advisable.
- Patients in the critical category, would need, in addition to the markers mentioned in the above categories, serum IL-6 levels and serial lactate levels.
- To monitor hospitalized patients on therapy, CBC and CRP should be repeated 48 to 72 h after admission or earlier. Serum ferritin cannot be recommended to monitor response to therapy based on current evidence.
- For children with suspected MIS-C, many investigations may be expensive, not easily available and time consuming. A tiered testing strategy can be employed provided the patient's clinical condition permits (75, 89). Complete blood count, CRP, electrolytes and liver function tests form a part of testing of all febrile children admitted to the hospital. In the presence of “elevated ESR and/or CRP and at least 1 other suggestive laboratory feature (lymphopenia, neutrophilia, thrombocytopenia, hyponatremia, or hypoalbuminemia),” the next tier of investigations (ferritin, d-dimer, PT, PTT, fibrinogen, troponin I, NT-pro-BNP) should be performed (75). Cytokine levels (IL-6, IL-10) may support the diagnosis and are not required to determine treatment. IL-6 levels may be considered in treatment of refractory cases when specific therapies like tocilizumab are considered. SARS-CoV-2 serology has been reported positive more often than PCR testing and both should be sent to evaluate the epidemiologic link to the infection. Response to therapy can be monitored by repeating CBC and CRP 24 to 48 h after therapy.

## Conclusion

COVID-19 is a heterogeneous disease spectrum with manifestations varying with age and presence of co-morbidities. Biomarkers will play a crucial role in early suspicion, diagnosis, monitoring, and recognition of complications, management and disposition of patients. Each of these components in turn can have crucial implications on the healthcare system and the administrative machinery, directly impacting patient care. Needless to say, clinical evaluation will be paramount at every step and biomarkers will need to be meaningfully integrated into bedside decision making. Biomarker panels rather than single biomarkers may provide more reliable information. Availability and cost issues cannot be ignored. It would be impossible for clinicians to consolidate and critically analyse the enormous data that is continuously added to the COVID-19 literature to extract practically useful information for the benefit of patients. National or regional guidelines which tailor the information available to suit the local population are essential.

## Author Contributions

MS performed the literature review and prepared the initial draft of the manuscript. MJ critically reviewed the manuscript and revised the initial draft. Both authors approved the final version.

## Conflict of Interest

The authors declare that the research was conducted in the absence of any commercial or financial relationships that could be construed as a potential conflict of interest.
